# Oligocentric Castleman disease: clinical characteristics and surgical outcomes from a single-centre retrospective study

**DOI:** 10.3389/fimmu.2025.1637792

**Published:** 2025-11-19

**Authors:** Yukai Duan, Xiang Gao, Siyu Qian, Renjie Hua, Honghan Qiao, Sijun Zhang, Feiyang Zong, Suxiao Li, Yunfei Song, Mingzhi Zhang, Qingjiang Chen, Meng Dong, Xudong Zhang

**Affiliations:** Department of Oncology, The First Affiliated Hospital of Zhengzhou University, Zhengzhou, Henan, China

**Keywords:** Castleman disease, treatment options, survival analysis, Oligocentric Castleman disease, surgery

## Abstract

**Introduction:**

Oligocentric Castleman Disease (OCD), a distinct subtype of Castleman Disease (CD) intermediate between Unicentric (UCD) and idiopathic Multicentric (iMCD) forms, remains poorly characterised.

**Methods:**

This study retrospectively analysed the clinical characteristics, treatment, and prognosis of 100 CD patients (63 UCD, 37 OligoCD).

**Results:**

Compared with UCD, OCD patients had a higher proportion of mixed type (Mixed-CD) histology and elevated CRP/ESR levels, along with significantly poorer Progression-Free Survival (PFS) (P = 0.0067). Within the OCD cohort, debulking surgery alone or combined chemotherapy achieved an 80.0% Complete Response (CR) rate; plasmacytic type(PC-CD), non-contiguous lesions, involvement of ≥3 regions, failure to achieve CR after initial treatment, and elevated baseline inflammatory markers were significant predictors of inferior PFS. Exploratory subgroup analysis divided the OCD cohort into asymptomatic and high-inflammatory groups with significantly different PFS (P = 0.042). The rate of progression to iMCD among surgically managed asymptomatic OCD patients in our study was similar to that in a large aMCD cohort predominantly managed with ‘watch-and-wait’, suggesting ‘active surveillance’ might be a more appropriate initial strategy for asymptomatic OCD. For the high-inflammatory subgroup, characterised by higher PC-CD rates and more widespread disease distribution despite effective symptom control with surgery, the post-operative relapse risk was higher.

**Discussion:**

In conclusion, debulking surgery is effective for alleviating symptoms in OCD but may be unnecessary for asymptomatic patients; factors associated with a hyper-inflammatory state predict relapse, underscoring the need for careful treatment planning and exploration of novel therapeutic strategies for this high-risk subgroup.

## 1 Introduction

Castleman disease (CD) is a group of rare, heterogeneous lymphoproliferative diseases characterised by distinctive histopathological features and the pathologist Castleman first reported this disease in 1956 (1). The exact pathogenesis of CD is still unknown, though interleukin-6 (IL-6) is believed to be a potential driving factor, and some cases are closely linked to human herpesvirus-8 (HHV-8) infection. Pathologically, CD can be classified into hyaline vascular type (HV-CD), plasmacytic type (PC-CD), and mixed type (Mixed-CD). Based on the number of affected lymph node regions, it is classified into unicentric CD (UCD) and multicentric CD (MCD).

UCD manifests as a solitary enlarged mass with laboratory findings mostly normal and usually without systemic symptoms (2). When surgical resection is feasible, complete removal yields excellent outcomes. For unresectable disease, locoregional therapies, such as radiotherapy or vascular embolization, may be employed depending on the patient’s disease status (3, 4). A condition known as UCD with MCD-like inflammatory state (UCD-MIS) occurs in a subset of UCD patients who manifest systemic inflammatory symptoms; notably, these manifestations can endure post-resection in some individuals (5, 6).

The progression of MCD is systemic and progressive, presenting with lymphadenopathy in multiple lymph node regions. Based on whether or notHHV-8 infection is present, it is classified into iMCD and HHV-8-associated MCD. Diagnosis of iMCD requires histopathological features consistent with CD, ≥2 enlarged lymph nodes, and at least 2 of 11 minor diagnostic criteria, with at least 1 being a laboratory abnormality (2). Currently, iMCD is classified into three main clinical subtypes, including iMCD-TAFRO(thrombocytopenia, anasarca, fever, reticulin fibrosis or renal insufficiency, and organomegaly), iMCD-IPL(idiopathic plasmacytic lymphadenopathy) and iMCD-NOS (Not Otherwise Specified) (7–12).

However, there has long been a nosological void between the localised disease of UCD and the systemic inflammatory cascade of iMCD. The formal proposition of Oligocentric Castleman Disease (OCD) has filled this gap, signifying a further refinement in our understanding of the CD spectrum (13).

OCD is defined as a distinct clinicopathological entity, intermediate between UCD and iMCD. At its core, the condition is defined by: confirmed CD histopathology; radiological evidence of lymphadenopathy affecting ≥2 nodal regions(typically defined as 2-5) (thereby not meeting the ‘single-centre’ definition of UCD); and a clinically indolent phenotype akin to that of UCD, failing to meet the full diagnostic criteria for symptomatic iMCD (i.e., presenting with fewer than two minor criteria) and disease confined to one side of the diaphragm (3, 13, 14).

It is estimated that OCD accounts for approximately 7.8% of all CD cases (13). Owing to its recent definition, there are no standardised treatment guidelines. Strategies involving consolidative radiotherapy after initial systemic treatment have been explored (15). Historically, these patients were often inconsistently categorised as either UCD or iMCD, which posed a significant therapeutic dilemma. The rarity of OCD has not only limited published studies to small sample sizes but also causes it to be frequently conflated with UCD in clinical practice, owing to the significant overlap in their clinical features. Therefore, we undertook this study to describe the clinical characteristics, treatment regimens, and prognosis of OCD, compare it with UCD to explore its unique characteristics and potential therapeutic strategies.

## 2 Subjects and methods

### 2.1 Patients

A total of 276 treatment-naïve patients with Castleman Disease (CD), diagnosed at our institution between November 2013 and April 2023, were identified through the information system of The First Affiliated Hospital of Zhengzhou University.

The initial classification yielded 81 cases of UCD, 39 cases of OCD, and 156 cases of iMCD. In this study, all cases underwent a central review by two expert pathologists upon patient enrolment to ensure the exclusion of cases not meeting the classic diagnostic criteria for CD. We ultimately identified a final cohort consisting of 74(29.8%) cases of UCD, 38(15.3%) cases of OCD, and 136(54.8%) cases of iMCD. The present study focuses on the UCD and OCD subtypes due to their greater clinical similarity. The study was approved by the Ethics Committee of the First Affiliated Hospital of Zhengzhou University (Ethical Approval No. 2022-KY-0869-001).

### 2.2 Procedures

**Figure 1:**
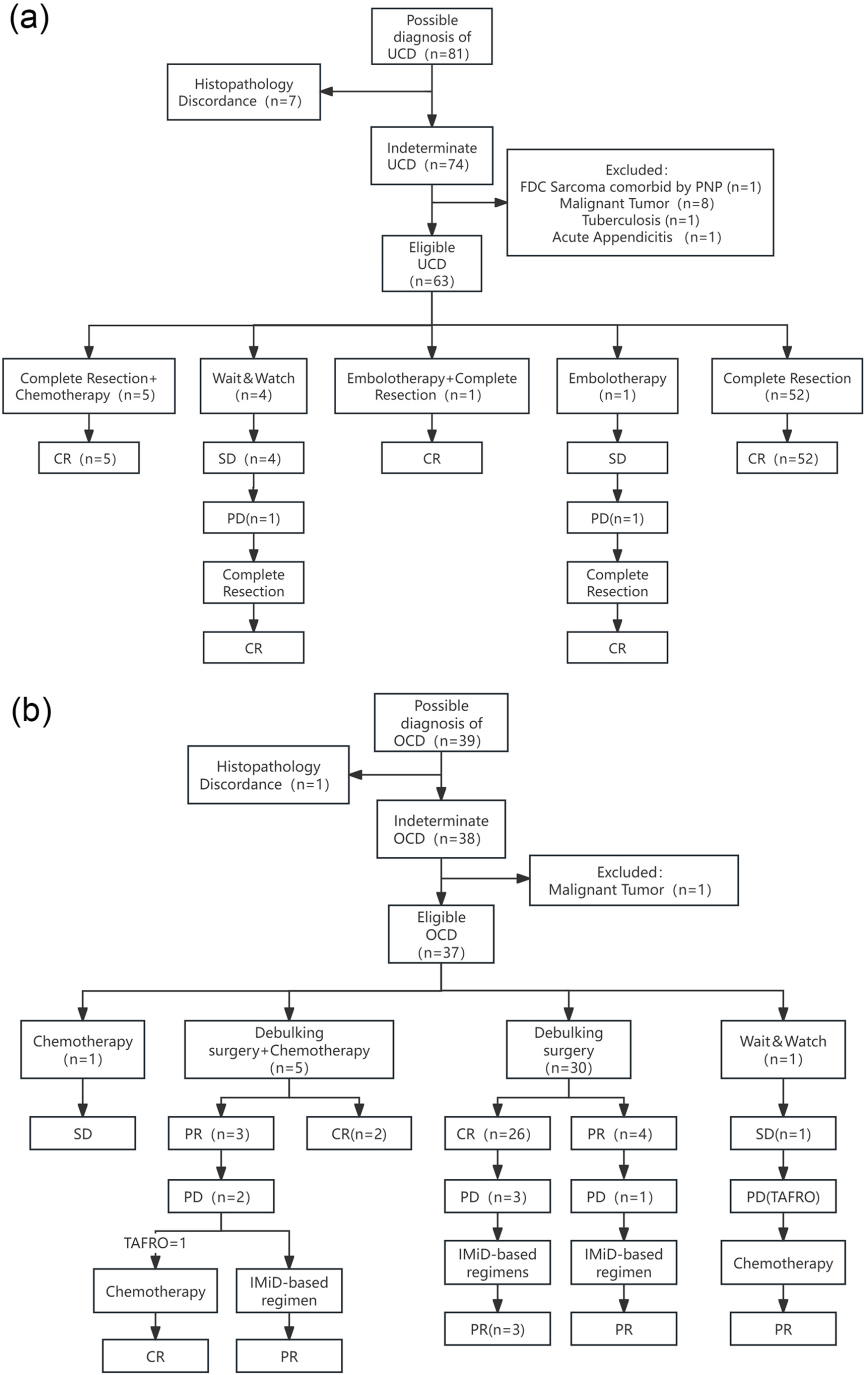
Enrollment procedure, treatment regimen, and response of UCD group **(a)** and OCD group **(b)**. FDC Sarcoma, Follicular Dendritic Cell Sarcoma; PNP, Paraneoplastic Pemphigus; IMiD-based regimen, regimen consisting of a thalidomide- or lenalidomide-based immunomodulatory agent, administered with or without a corticosteroid; TAFRO, a syndrome of thrombocytopenia, anasarca, fever, reticulin fibrosis or renal insufficiency, and organomegaly, a severe subtype of iMCD.

The diagnoses of UCD and OCD were re-confirmed by reviewing admission records, imaging reports, and laboratory tests. The imaging information of the cases was re-evaluated to determine whether the disease was unicentric or oligocentric. Cases with concurrent tumours, infections, or autoimmune diseases were excluded from both groups, as these conditions might accompany CD lymph node pathological changes. Ultimately, 63 cases were included in the UCD group and 37 in the OCD group (**Figures 1a, b**). Demographic, clinical, laboratory, and treatment-related data, including age, gender, presenting symptoms, physical examination findings, imaging and laboratory tests, histopathology, treatment strategies, and outcomes, were extracted from the patients’ medical records.

### 2.3 Efficacy assessment

In the absence of standardised response criteria for OCD, our efficacy assessment was based on the established framework for iMCD, which includes laboratory results: C-reactive protein (CRP), hemoglobin (HGB), albumin, glomerular filtration rate (GFR), systemic symptoms (fatigue, anorexia, fever, weight loss), and the largest diameter of enlarged lymph nodes.① Complete Response (CR) is defined as normal laboratory test results and systemic symptoms returning to normal.② Partial Response (PR) is defined as improvement in 4 systemic symptoms, laboratory results improving by more than 50%, and a reduction of more than 50% in the largest diameter of the enlarged lymph nodes.③ Progressive Disease (PD) is defined as a worsening of laboratory test results by more than 25%, any of the 4 systemic symptoms related to inflammation worsening, an increase of more than 25% in the largest diameter of enlarged lymph nodes, or relapse.④ Stable Disease (SD) is defined as the absence of partial or complete remission or progressive disease (16).

### 2.4 Follow-up results

Follow-up was conducted by telephone, reviewing patients’ inpatient records, and outpatient follow-up records, with the follow-up cutoff date of May 31, 2025. The median follow-up time was 67.0 (25-141) months. The primary endpoints for this study were Overall Survival (OS) and Progression-Free Survival (PFS).

### 2.5 Statistical analysis

Descriptive statistics were used to summarise case characteristics. Clinical data were compared between the UCD and OligoCD groups using the chi-squared test, corrected chi-squared test, Fisher’s exact test, independent samples t-test, or Mann-Whitney U test, as appropriate. OS and PFS was estimated using the Kaplan-Meier method. Univariate analysis for PFS involved group comparisons using the log-rank test and calculation of Hazard Ratios (HRs) with 95% Confidence Intervals (CIs) using univariate Cox proportional hazards models. A P-value of less than 0.05 was considered statistically significant. All statistical analyses were performed using SPSS version 25.0 (IBM, Armonk, NY) and R software version 4.5.1.

## 3 Results

### 3.1 Baseline characteristics

**Figure 2:**
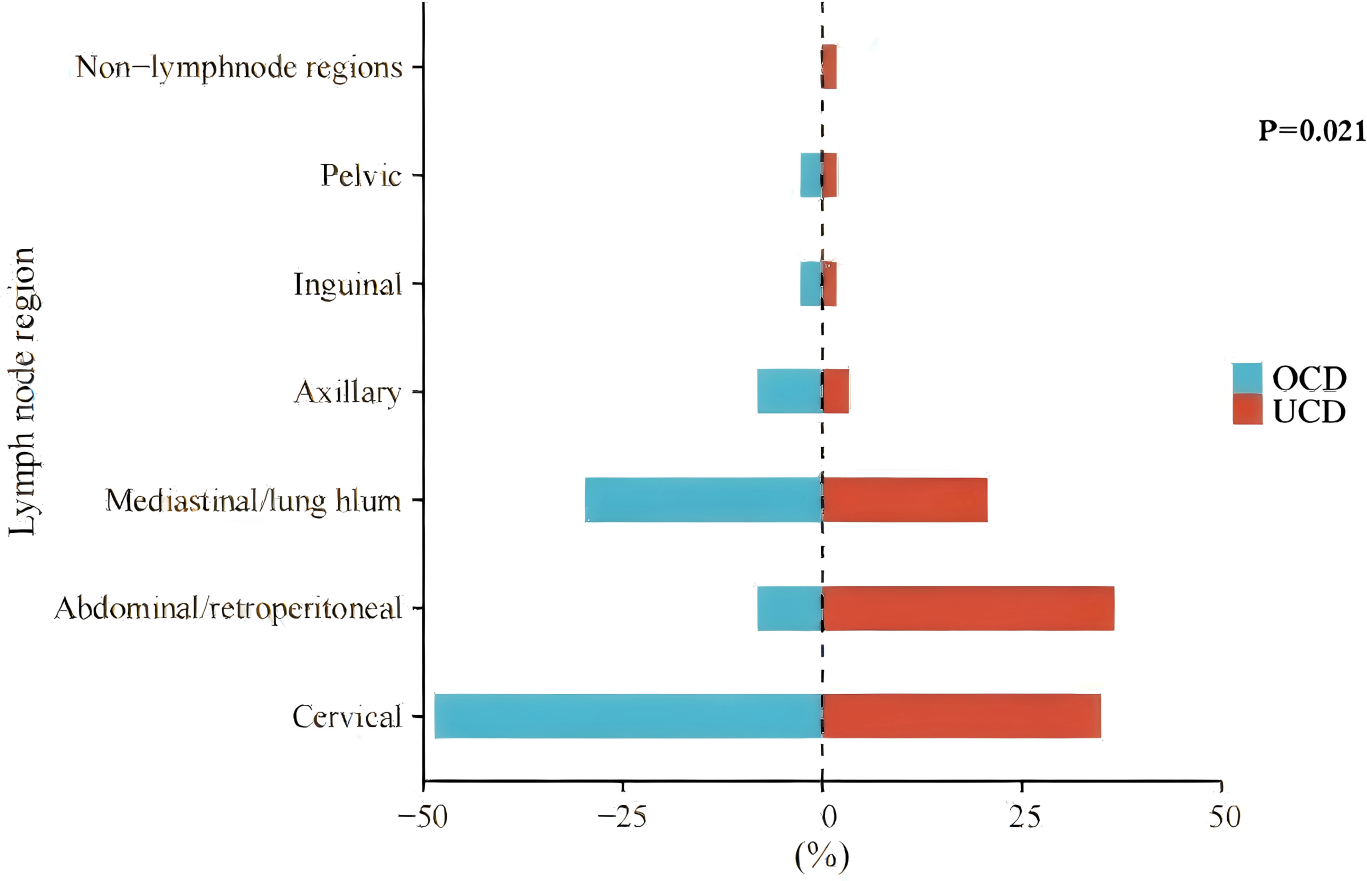
The distribution of the lymph node region affected by the largest lymph node (LNRALN) in UCD and OCD. Non-lymphnode regions, disease involving an extranodal site.

There is no significant difference in gender or age distribution was observed between the two groups. In the UCD group, the maximum diameter of the enlarged lymph nodes (MDLN) was 5.55 ± 2.46 cm, and the lymph node region affected by the largest lymph node (LNRALN) was most commonly found in the abdomen/retroperitoneum (36.5%). In the OCD group, the MDLN was 5.14 ± 1.66 cm. The LNRALN was most commonly found in the neck (48.6%). There was a significant difference in LNRALN between the two groups (p=0.021) (**Table 1**, **Figure 2**). All patients presented with disease on one side of the diaphragm.

**Table 1:** Clinical features and pathological classification of the two groups.

Characteristic	UCD(n=63)	OCD(n=37)	*P*
Age, mean ± SD, y	39.2 ± 16.5	35.4 ± 14.1	0.242
Sex, n(%)			0.907
Male	28(44.4)	16(43.2%)	/
Female	35(55.6)	21(56.8%)	/
Histopathology, n (%)			***0.003***
HV-CD	57(90.5)	23(62.2%)	***0.001***
Mixed-CD	4(6.3)	10(27.0%)	***0.004***
PC-CD	2(3.2)	4(10.8%)	0.190
LNRALN, n(%)			***0.021***
Cervical	22(34.9)	18(48.6)	/
Abdominal/retroperitoneal	23(36.5)	3(8.1)	/
Mediastinal/lung hlum	13(20.6)	11(29.7)	/
Axillary	2(3.2)	3(8.1)	/
Inguinal	1(1.6)	1(2.7)	/
Pelvic	1(1.6)	1(2.7)	/
Non-lymphnode regions	1(1.6)	0(0.0)	/
MDLN (cm)	5.55 ± 2.46	5.14 ± 1.66	0.372
Presenting Complaint, n (%)			0.201
Systemic symptoms^a^	3(4.8)	2(5.4)	
Local symptoms^b^	13(20.6)	7(18.9)	
Incidental finding^c^	47(74.6)	28(75.7)	

LNRALN, lymph node region affected by the largest lymph node; MDLN, the maximum diameter of the enlarged lymph nodes. ^a^Including night sweats, fever (>38°C), weight loss (≥10% over 6 months), or fatigue (affecting instrumental activities of daily living); ^b^Clinical manifestations caused by the compression of local tissues or organs by the bulk; ^c^Discovered incidentally or during routine health screening. Bold values indicate statistical significance (P < 0.05).

There was a significant difference in pathological classification between the two groups (p=0.003), with the HV-CDtype being more prevalent in the UCD group (90.5% vs 62.2%) (p=0.001); the mixed type was more prevalent in the aMCD group (27.8% vs 6.3%) (p=0.004) (**Table 1**).

We compiled the reasons for patients’ hospital admission and consultation, defining systemic symptoms. The reasons for presentation did not differ significantly between the two groups; in both cohorts, the majority of cases were diagnosed incidentally(74.6% vs 75.7%), while patients presenting with systemic symptoms represented a minority(4.8% vs 5.4%). (**Table 1**) Compared with the UCD group, the OCD group demonstrated a significantly higher proportion of cases with elevated levels of ESR and CRP (26.7% vs 5.7%, 30.0% vs 3.8%, respectively), with all differences being statistically significant (**Table 2**).

**Table 2:** Laboratory examination results of the two groups.

Characteristic	UCD		OCD		
	*n*	*n*(%)	*n*	*n*(%)	*P*
Constitutional symptoms^a^	63	3(4.8)	37	2(5.4)	1.000
Hepatomegaly and/or splenomegaly	63	3(4.8)	37	2(5.4)	1.000
Fluid accumulation^b^	63	8(12.7)	37	2(5.4)	0.315
Anemia^c^	63	8(12.7)	37	5(13.5)	1.000
Abnormal PLT^d^	63	5(7.9)	37	2(5.4)	0.942
Albumin<35, g/l	63	1(1.6)	37	1(2.7)	1.000
eGFR<60.00, mL/min per 1.73 m^2^	63	0(0)	37	0(0)	/
ESR>15, mm/h	52	2(3.8)	30	8(26.7)	***0.007***
CRP>10.00, mg/L	52	2(3.8)	30	9(30.0)	***0.003***
IgG>17.00, g/L	4	0(0)	4	2(50.0)	/
IL-6>5.9, pg/mL	4	1(25.0)	3	1(33.3)	/
Lactate dehydrogenase(LDH)>280, U/L	63	3(4.8)	37	1(2.7)	1.000
Beta-2 microglobulin(β2M)>2.5, mg/L	63	0(0)	37	1(2.7)	0.370

^a^Night sweats, fever (>38°C), weight loss, or fatigue (≥2 CTCAE lymphoma score for B-symptoms).

^b^Edema, anasarca, ascites, or pleural effusion.

^c^Hemoglobin: <12 g/dL in males, <11 g/dL in females.

^d^Thrombocytopenia (PLT<100 k/μL) or thrombocytosis (PLT>350 k/μL). Bold values indicate statistical significance (P < 0.05).

### 3.2 Characteristics of lymph node involvement in aMCD group

In the OCD cohort, 23(63.9%) cases involved only two lymph node regions, while 14 cases (36.1%) involved ≥3 lymph node regions.

To assess the internal heterogeneity of disease activity, 9 (24.3%) patients underwent 18F-Fluorodeoxyglucose Positron Emission Tomography/Computed Tomography (PET-CT) scans. The difference in Standard Uptake Value Maximum (SUVmax) between the largest lymph node and the other lymph node with the highest metabolic activity was not statistically significant (6.40 vs. 3.94) (t=1.836, P = 0.104).

Furthermore, to assess the heterogeneity of tumour bulk, a paired-samples t-test was performed comparing the Maximum Diameter of the Enlarged Lymph Nodes (MDLN) to the diameter of the second-largest involved lymph node in the OCD cohort (N = 37). The analysis revealed a highly statistically significant difference in size between the two lesion groups (MDLN, 5.14 ± 1.66cm vs. second-largest, 1.76 ± 0.44cm; t=13.281, P<0.001).

### 3.3 Treatment strategy and response

#### 3.3.1 Treatment strategies and outcomes for UCD

Complete Resection Alone (n=52, 82.5%): 52 cases received complete surgical resection as the sole first-line therapy, all of whom achieved a CR.

Complete Resection + Adjuvant Chemotherapy (n=5, 7.9%): 5 cases received adjuvant chemotherapy following complete resection, all achieving CR.

Interventional and Combined Therapy (n=2, 3.2%): 2 cases were ineligible for upfront resection due to large tumour burden. One case initially received arterial embolisation as first-line therapy, but the lesion did not shrink; mass enlargement was noted during subsequent observation, and the patient subsequently underwent complete resection, remaining in CR post-operatively. The other case successfully underwent complete resection after embolisation (to reduce surgical risk) and achieved CR.

Watch-and-Wait (n=4, 6.3%): 4 cases were managed with a watch-and-wait strategy, all maintaining SD during follow-up. One case experienced disease progression 34 months after initial diagnosis and subsequently underwent surgical treatment, remaining in CR post-operatively.

Resolution of Symptoms and Laboratory Abnormalities: Among all UCD cases, the 15 cases who presented at diagnosis with either laboratory abnormalities or systemic symptoms achieved complete resolution of both symptoms and indicators following treatment regimens that included surgical resection.

#### 3.3.2 Treatment strategies and outcome for OCD

Post-operative Response and Relapse

The surgical approach for all patients was not a radical complete resection. Instead, it involved a targeted resection of what were considered the primary lesions, determined by a comprehensive clinical evaluation incorporating imaging findings such as PET-CT. Following debulking surgery, 86.7% of cases (26 of 30) had normalisation of laboratory abnormalities, and any remaining enlarged lymph nodes gradually resolved. Of these responders, 11.5% (3 of 26 cases) subsequently relapsed, presenting with isolated lymphadenopathy without associated laboratory abnormalities. An incomplete response was noted in 13.3% of cases (3 of 30); 1 case had incomplete resolution of anaemia, while 2 cases were assessed as having less than a PR due to the persistence of other enlarged lymph nodes. 1 of these 3 cases later relapsed, also presenting with lymphadenopathy without abnormal laboratory findings (**Figure 1b**).

Outcomes for Other Management Strategies

Adjuvant Chemotherapy 5 cases (13.5% of 37) received conventional chemotherapy following debulking surgery. In 60% of these (3 of 5 cases), lymph nodes in other regions failed to resolve, and 2 of these 3 subsequently progressed after first-line treatment. 1 case developed recurrent pulmonary infections post-chemotherapy and was re-hospitalised 3 months after its completion with a diagnosis of TAFRO syndrome; a CR was achieved after treatment with high-dose methylprednisolone and lenalidomide (**Figure 1b**).

Chemotherapy Alone: 1 case (2.7% of 37) received chemotherapy as a single modality, after which the enlarged lymph nodes did not resolve (**Figure 1b**).

Watch-and-Wait: 1 case was managed with a watch-and-wait approach and was admitted 25 months later with a pulmonary infection. At that point, the disease had progressed and was diagnosed as TAFRO syndrome. Disease control was achieved following a cyclophosphamide, thalidomide, and prednisone regimen, which was followed by 4 cycles of R-CHOP for consolidation. However, the lymphadenopathy did not fully resolve, and the best response was a PR (**Figure 1b**).

Management of Isolated Nodal Relapse

**Figure 3:**
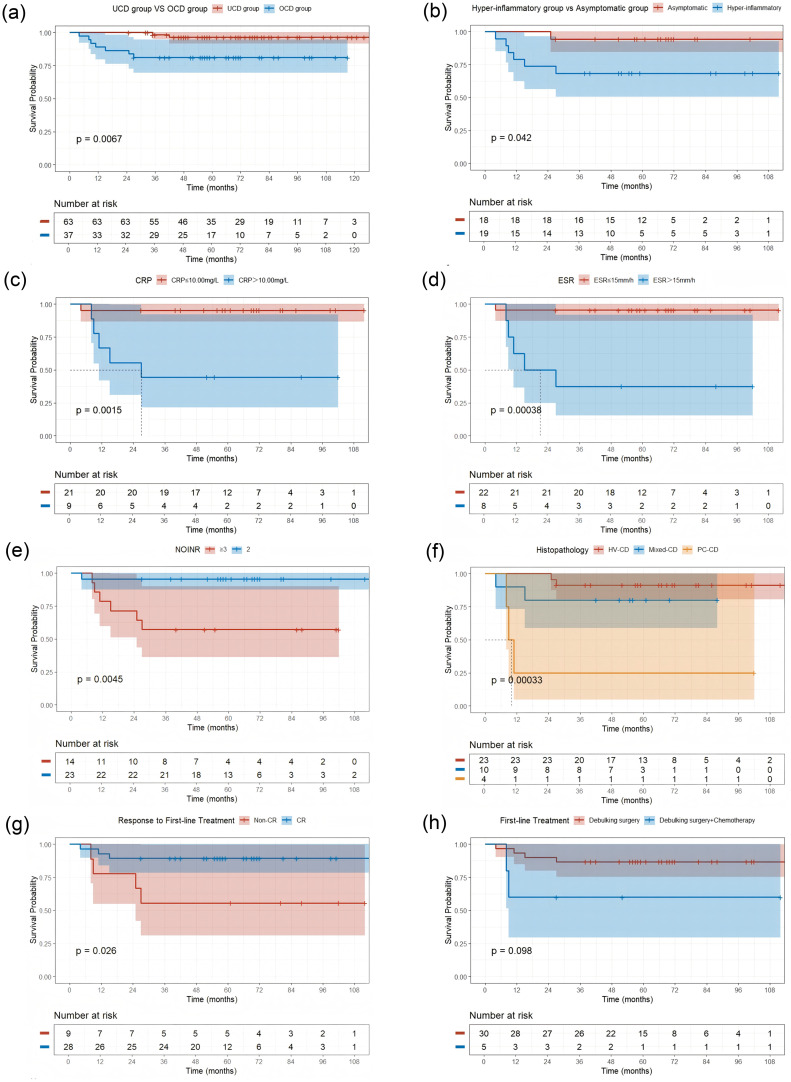
Kaplan-Meier Curves for Progression-Free Survival (PFS) by Different Factors **(a)** UCD group vs OCD group (P = 0.0067); **(b)** Hyper-inflammatory group vs Asymptomatic group (P = 0.042); **(c)** Stratified by C-reactive protein (CRP) level (P = 0.0015); **(d)** Stratified by elevated erythrocyte sedimentation rate (ESR) level (P = 0.00038); **(e)** Stratified by number of involved nodal regions (NOIOR) (P = 0.0045); **(f)** Stratified by Histopathological subtype (P = 0.00033); **(g)** Stratified by Response to First-line Treatment (Non-CR vs CR) (P = 0.026); **(h)** Stratified by First-line Treatment type (Debulking surgery vs Debulking surgery + Chemotherapy) (P = 0.098). Abbreviations: Non-CR, failure to achieve a complete response; CR, complete response.

Notably, all cases who experienced an isolated nodal relapse achieved disease control with a regimen based on an immunomodulatory drug (IMiD), such as thalidomide or lenalidomide. These cases have remained free of relapse or progression at long-term follow-up (**Figures 1b**, **3**).

Impact on Systemic Abnormalities

Among all OCD cases, 94.7% (18 of 19) of those presenting with abnormal laboratory results or systemic symptom experienced normalisation following debulking surgery. Among the 2 cases with hepatosplenomegaly, organ size normalised in 1 case following the procedure.

In the UCD group, 58 cases who received either complete surgical treatment or combined complete surgical treatment all achieved CR, resulting in an overall response rate (ORR) of 100%. In the OCD group, of the 35 cases who underwent either surgery alone or combined surgery, CR was achieved in 28 cases (80%), while PR was achieved in the remaining 7 cases (20%). A statistically significant difference in CR rates was observed between the UCD and OCD groups (P = 0.001).

### 3.4 Survival analysis

The median follow-up time was 67.0 (25-141) months. At the end of the follow-up period, no deaths occurred, except in cases lost to follow-up. Consequently, the primary analytical focus shifts to PFS as a more clinically relevant endpoint for assessing disease-related morbidity and control.

We performed a univariate analysis using the Kaplan-Meier method and the log-rank test to identify factors at diagnosis that were predictive of PFS. The results, revealed several prognostic indicators. Histopathological subtype was a key determinant of outcome. While the Mixed-CD type was not significantly associated with a poorer PFS (HR 2.62; 95% CI 0.37–18.6; P = 0.3), PC-CD had a profound and statistically significant negative impact on PFS (HR, 17.6; 95% CI 2.78–111; P = 0.002) (**Table 3**, **Figure 3f**).

**Table 3:** Univariate regression analysis for PFS in OCD group.

Characteristic	N(n%)	HR	95%CI	P-value
Male	16/37(43.2%)	0.93	0.21,4.18	>0.9
Mixed-CD	10/37(27.0%)	2.62	0.37,18.6	0.3
PC-CD	4/37(10.8%)	17.6	2.78,111	***0.002***
COINR Non-contiguous	7/37(18.9%)	20.9	3.92,112	<0.001
NOIOR≥3	14/37(37.8%)	11.4	1.37,95.3	***0.024***
Debulking surgery + Chemotherapy	5/37(13.5%)	3.81	0.69,21.0	0.12
Non-CR	9/37(24.3%)	4.7	1.05,21.0	***0.043***
CRP>10.00mg/L	9/30(30.0%)	14.3	1.65,123	***0.016***
ESR>15mm/h	8/30(26.7%)	17.6	2.03,152	***0.009***
Supradiaphragmatic disease	32/37 (86.5%)	0.86	0.10,7.18	0.9
Anaemia	5/37 (13.5%)	2.58	0.50,13.3	0.3

COINR, contiguity of involved nodal regions; NOIOR, number of involved nodal regions; Non-CR, failure to achieve a complete response to first-line therapy; Anaemia, haemoglobin<12 g/dL in males,<11 g/dL in females. Bold values indicate statistical significance (P < 0.05).

Factors related to the anatomical burden and distribution of disease were also associated with progression. Specifically, non-contiguous nodal involvement(COINR Non-contiguous) (HR 20.9; 95% CI 3.92–112; P < 0.001), and the involvement of three or more nodal regions(NOIOR≥3) (HR 11.4; 95% CI 1.37–95.3; P = 0.024) were all highly significant predictors of a shorter PFS (**Table 3**, **Figure 3e**).

Furthermore, a hyper-inflammatory state at diagnosis, manifested by elevated systemic inflammatory markers, was also significantly associated with a poor prognosis. This was evidenced by elevated levels of CRP (>10.00 mg/L: HR 14.3; 95% CI 1.65–123; P = 0.016) and ESR (>15 mm/h: HR 17.6; 95% CI 2.03–152; P = 0.009). Finally, failure to achieve a CR to initial therapy was also a significant predictor of subsequent progression (HR 4.7; 95% CI 1.05–21.0; P = 0.043) (**Table 3**, **Figures 3c, d, g**).

We attempted to construct a multivariate Cox proportional hazards model to identify independent prognostic factors. However, due to the small number of endpoint events in this cohort (only 6 progression events among 37 patients), the model was statistically unstable and demonstrated the phenomenon of complete separation. This issue precluded the calculation of valid hazard ratios, rendering the multivariate analysis uninformative.

### 3.5 Comparison with existing cohorts

To contextualise our findings within the evolving nosology of Castleman disease, we compared the clinicopathological features and outcomes of our OCD cohort with two recently described and highly relevant patient populations: the initial OCD cohort described by Pierson et al. (13), and the asymptomatic multicentric Castleman disease (aMCD) cohort reported by Zhang et al. (**Table 4**).

**Table 4:** Comparison with the existing literature.

Characteristic	OCD cohort(Pierson et al.) (13)	aMCD cohort (Zhang et al.) (17)	Our OCD cohort
Patients (N)	14	114	37
Baseline characteristics			
Mean/Median Age	34.0 (Mean)	45.5 (Median)	35.4 (Mean)
Female (%)	71.40% (10/14)	47.40% (54/114)	56.80% (21/37)
Distribution of Involved Nodal Regions	Exclusively unilateral to the diaphragm (100%)	24 (21.1%) with bilateral involvement	Exclusively unilateral to the diaphragm (100%)
Histopathological subtype (%)			
- HV-CD	78.60% (11/14)	48.2% (55/114)	62.20% (23/37)
- Non-HV-CD	21.40% (3/14)	51.8% (59/114)	37.80% (14/37)
Treatment information			
Watch-and-Wait	0.00%	62.3% (71/114)	2.7% (1/37)
Surgery-based Treatment	>70%	Not Applicable	94.6% (35/37)
First-line Systemic Therapy	A minority of cases	37.7% (43/114)	2.7% (1/37)
Treatment outcomes			
Watch-and-Wait	Not Applicable	4.2% (3/71) progressed to iMCD	1(100%) progressed to iMCD
Surgery-based Treatment	40% (Nodal response rate)	Not Applicable	86.7% (CR)
First-line Systemic Therapy	Insufficient data	6.9% (3/43) of cases progressed to iMCD	Only 1 receiving chemotherapy as a single modality failed to achieve a response;
Overall Progression Rate	35.7% (5/14)	5.3% (6/114)	18.9% (7/37)
Progression to iMCD	Insufficient data	5.3% (6/114)	Overall 5.4% (2/37) Asymptomatic group 5.6%(1/18) Hyper-inflammatory group 5.3%(1/19)

Our study cohort (N = 37) is substantially larger than the seminal cohort (N = 14) originally used by Pierson et al. to define the OligoCD entity. While key demographic features such as mean age were comparable (35.4 vs. 34.0 years), critical differences exist in disease distribution and pathology. Consistent with the cohort described by Pierson and colleagues, our patients also presented with disease exclusively confined to one side of the diaphragm. A critical difference, however, was observed in the histopathology. Our cohort included a significant proportion of cases with the PC-CD subtype (10.8%), whereas this variant was entirely absent from the Pierson cohort (0.0%).

A comparison with the large, multicentric cohort of 114 aMCD patients described by Zhang et al. provides a robust benchmark for indolent yet multicentric disease. Despite these differing management philosophies, the most striking finding was the remarkably similar rate of progression to iMCD. In our cohort, 2 of 37 cases (5.4%) progressed to iMCD during follow-up, a proportion nearly identical to that in the cohort by Zhang et al., where 6 of 114 cases (5.3%) underwent a similar transformation.

### 3.6 Exploratory subgroup analysis

Given the significant heterogeneity observed in PFS during the univariate analysis, we conducted an exploratory subgroup analysis to stratify the OCD cohort into more clinically and biologically homogeneous subgroups. Cases were categorised into either an ‘asymptomatic group’, defined by the absence of systemic symptoms and normal inflammatory markers (analogous to the aMCD entity), or a ‘high-inflammatory group’, which included cases with either abnormal laboratory parameters, primarily elevated inflammatory markers. or systemic symptoms that did not meet the formal diagnostic criteria for iMCD. The core finding of this analysis was the profound and highly statistically significant difference in PFS between these two subgroups. Kaplan-Meier analysis revealed that patients in the high-inflammatory group had a markedly poorer PFS compared with those in the asymptomatic group (log-rank test, P = 0.042) (**Figure 3b**).

Subsequent analysis of the clinicopathological characteristics of these subgroups revealed a distinct set of high-risk features defining the ‘high-inflammatory’ phenotype. A significant difference was observed in the distribution of histopathological subtypes between the two groups (overall P = 0.018). This difference was primarily driven by a significantly lower prevalence of the indolent HV-CD subtype in the high-inflammatory group (P = 0.017). Notably, all four PC-CD cases in the cohort were found within the high-inflammatory group, although this specific comparison did not reach statistical significance (P = 0.105) (**Table 5**).

**Table 5:** Characteristics used for exploratory subgroup analysis.

Characteristic	Asymptomatic group	Hyper-inflammatory group	*P*
Age, mean ± SD, y	31.89±13.05	38.79±14.66	0.140
Sex,n(%)			0.515
Male	9	7	
Female	9	12	
Histopathology, n (%)			***0.018***
HV-CD	15	8	***0.017***
Mixed-CD	3	7	0.269
PC-CD	0	4	0.105
COINR			***0.008***
Contiguous	18	12	
Non-contiguous	0	7	
NOIOR			***0.017***
2	15	8	
≥3	3	11	
Involved region			1.000
Supradiaphragmatic	16	16	
Infradiaphragmatic	2	3	

COINR, contiguity of involved nodal regions; NOIOR, number of involved nodal regions. Bold values indicate statistical significance (P < 0.05).

Significant differences were also noted in the pattern of nodal involvement. The high-inflammatory group was characterised by a more widespread and systemic distribution of disease, with a significantly higher rate of non-contiguous nodal involvement (P = 0.008) and a greater number of involved nodal regions (P = 0.017) (**Table 5**).

## 4 Discussion

To the best of our knowledge, this represents the largest single-centre study to date on OCD. This study is a retrospective analysis of 37 cases with OCD, aimed at exploring the clinical heterogeneity and optimal management strategies for this emerging subtype. At the end of follow-up, no deaths were observed, confirming the indolent clinical nature of OCD with respect to mortality and supporting the use of PFS as the key endpoint for assessing disease control. Our core finding is that despite its overall indolent behaviour, OCD is a markedly heterogeneous disease. This study successfully stratifies cases with OCD into a low-risk ‘asymptomatic’ subgroup and a high-risk ‘high-inflammatory’ subgroup, the latter of which is defined by distinct clinicopathological features and is predictive of a higher risk of relapse.

While our asymptomatic OCD subgroup demonstrated excellent outcomes following surgery, was this success truly attributable to the intervention? A comparison with the large aMCD cohort, which shares a nearly identical definition with our asymptomatic subgroup, is particularly revealing. In that cohort, “watch-and-wait” was the predominant strategy, yet the rate of progression to symptomatic iMCD (5.3%) was remarkably similar to that of our surgically-treated cohort (5.4%) (17). This striking parallel not only suggests that a small subset of cases harbors an inherent biological risk of progression that is not entirely mitigated by the initial choice of therapy, but it also strongly implies that for asymptomatic cases with a confirmed diagnosis, active surveillance is likely a more beneficial approach than immediate invasive surgery.

When surgical intervention is clinically indicated, precisely delineating the extent of resection to balance efficacy against risk is of paramount importance. Our study provides potential anatomical evidence for a targeted surgical strategy: in our cohort, the lymph node with the MDLN was significantly larger than the next largest lymph node. This significant size discrepancy biased our clinical decision-making towards a limited surgical strategy of precisely resecting this dominant lesion, rather than performing an extensive lymph node dissection; this also explains why the majority of our cases were managed surgically. This surgical approach is not unique to our centre; all 14 OligoCD cases in the registry study by Pierson et al. also underwent lymph node excision (partial or complete) (13). By targeting this probable ‘driver’ centre, it may be possible to achieve an effective reduction in disease burden while minimising the surgical scope, thereby reducing the risk of long-term complications such as chronic lymphoedema. Due to the very small sample size of OCD cases receiving surgery combined with chemotherapy, our analysis was merely exploratory and lacks statistical significance. However, for a disease exhibiting a markedly indolent nature, chemotherapy appears to represent potential overtreatment.

A core observation from our study reveals a key paradox in the treatment of OCD: while surgery is highly effective at alleviating symptoms and normalising abnormal laboratory parameters, cases with a concurrent ‘broader’ disease state appear more prone to relapse post-operatively, even after achieving an initial CR. This risk of early relapse has been noted in previous reports on OCD (13), suggesting that although surgery removes the primary ‘local lesion-driven’, it may not eradicate the underlying aetiology of the disease. We therefore cautiously propose that this may reflect a regional ‘field effect’, potentially driven by deeper underlying factors, where the pathological process extends beyond individual nodal regions but has not yet developed into the systemic ‘spill-over’ characteristic of iMCD. This positions it between the surgically curable ‘ UCD cases with an MCD-like inflammatory state (UCD-MIS)’ entity (5) and the systemically-driven iMCD. This inherent risk of relapse raises an important clinical consideration: for cases identified at diagnosis as ‘high-inflammatory OCD’, adopting a well-tolerated, chemotherapy-free oral regimen as a first-line therapy could be a viable alternative. This is a strategy, however, that requires further exploration in larger, prospective studies.

Our study also offers insights for managing high-risk OCD cases, including those relapsing post-surgery. Univariate analysis confirmed that PC-CD histology, high anatomical burden (non-contiguous involvement or ≥3 regions), and a hyper-inflammatory state (elevated CRP/ESR) are strong predictors of relapse, findings consistent with established iMCD risk models (18). These factors effectively delineated our high-risk ‘high-inflammatory OCD’ subgroup. Critically, for patients relapsing after surgery in our cohort, second-line therapy with immunomodulatory drug (IMiD)-based regimens (thalidomide or lenalidomide) achieved good disease control, aligning with reported IMiD efficacy in refractory iMCD (19, 20). This suggests that prospectively exploring chemotherapy-free, IMiD-based regimens as adjuvant or even first-line therapy for high-risk OCD represents a promising future research avenue.

Our study is subject to several limitations. Its retrospective and single-centre design may introduce selection bias. Most importantly, the sample size is relatively limited; consequently, our conclusions—particularly those from the subgroup analyses and regarding the efficacy of second-line therapies—should be considered exploratory and require validation in larger, multicentre, prospective studies.

In conclusion, this study provides valuable single-centre data on the clinical heterogeneity of OCD as an emerging disease entity. Our findings suggest that a more prudent and individualised strategy for the management of OCD is required. For low-risk, asymptomatic cases, active surveillance may represent a reasonable option. For the high-inflammatory subgroup, the phenomenon of a high post-operative relapse risk suggests that while surgery can control symptoms, it may not eradicate the underlying pathological process. Ultimately, for cases harbouring high-risk features, our data provide a preliminary rationale for the future exploration of immunomodulatory-based systemic therapies.

## Data Availability

The raw data supporting the conclusions of this article will be made available by the authors, without undue reservation.

## References

[1] Castleman B Iverson L Menendez VP . Localized mediastinal lymphnode hyperplasia resembling thymoma. Cancer. (1956) 9:822–30. doi: 10.1002/1097-0142(195607/08)9:4<822::aid-cncr2820090430>3.0.co;2-4, PMID: 13356266

[2] Fajgenbaum DC Uldrick TS Bagg A Frank D Wu D Srkalovic G . International, evidence-based consensus diagnostic criteria for HHV-8-negative/idiopathic multicentric Castleman disease. Blood. (2017) 129:1646–57. doi: 10.1182/blood-2016-10-746933, PMID: 28087540 PMC5364342

[3] van Rhee F Oksenhendler E Srkalovic G Voorhees P Lim M Dispenzieri A . International evidence-based consensus diagnostic and treatment guidelines for unicentric Castleman disease. Blood Adv. (2020) 4:6039–50. doi: 10.1182/bloodadvances.2020003334, PMID: 33284946 PMC7724917

[4] Hematology Committee of Chinese Medical A. A . Hematological Oncology Committee of China Anti-Cancer and N. China Castleman Disease, The consensus of the diagnosis and treatment of Castleman disease in China. Chin J Hematol. (2021) 42:529–34. doi: 10.3760/cma.j.issn.0253-2727.2021.07.001, PMID: 34455738 PMC8408489

[5] Zhang MY Jia MN Chen J Feng J Cao XX Zhou DB . UCD with MCD-like inflammatory state: surgical excision is highly effective. Blood Adv. (2021) 5:122–8. doi: 10.1182/bloodadvances.2020003607, PMID: 33570636 PMC7805307

[6] Sarmiento Bustamante M Shyamsundar S Coren FR Bagg A Srkalovic G Alapat D . Ongoing symptoms following complete surgical excision in unicentric Castleman disease. Am J Hematol. (2023) 98:E334–7. doi: 10.1002/ajh.27065, PMID: 37635628 PMC10998479

[7] Zhang L Dong YJ Peng HL Li H Zhang MZ Wang HH . A national, multicenter, retrospective study of Castleman disease in China implementing CDCN criteria. Lancet Reg Health West Pac. (2023) 34:100720. doi: 10.1016/j.lanwpc.2023.100720, PMID: 37283978 PMC10240357

[8] Iwaki N Fajgenbaum DC Nabel CS Gion Y Kondo E Kawano M . Clinicopathologic analysis of TAFRO syndrome demonstrates a distinct subtype of HHV-8-negative multicentric Castleman disease. Am J Hematol. (2016) 91:220–6. doi: 10.1002/ajh.24242, PMID: 26805758

[9] Nishimura Y Fajgenbaum DC Pierson SK Iwaki N Nishikori A Kawano M . Validated international definition of the thrombocytopenia, anasarca, fever, reticulin fibrosis, renal insufficiency, and organomegaly clinical subtype (TAFRO) of idiopathic multicentric Castleman disease. Am J Hematol. (2021) 96:1241–52. doi: 10.1002/ajh.26292, PMID: 34265103 PMC9642098

[10] Takeuchi K . Idiopathic plasmacytic lymphadenopathy: A conceptual history along with a translation of the original Japanese article published in 1980. J Clin Exp Hematop. (2022) 62:79–84. doi: 10.3960/jslrt.22011, PMID: 35768240 PMC9353855

[11] Nishikori A Nishimura MF Nishimura Y Otsuka F Maehama K Ohsawa K . Idiopathic plasmacytic lymphadenopathy forms an independent subtype of idiopathic multicentric castleman disease. Int J Mol Sci. (2022) 23(18):10301. doi: 10.3390/ijms231810301, PMID: 36142213 PMC9499480

[12] Gao YH Liu YT Zhang MY Li SY Fajgenbaum DC Zhang L . Idiopathic multicentric Castleman disease (iMCD)-idiopathic plasmacytic lymphadenopathy: A distinct subtype of iMCD-not otherwise specified with different clinical features and better survival. Br J Haematol. (2024) 204:1830–7. doi: 10.1111/bjh.19334, PMID: 38356434 PMC11090736

[13] Pierson SK Brandstadter JD Torigian DA Bagg A Lechowicz MJ Alapat D . Characterizing the heterogeneity of Castleman disease and oligocentric subtype: findings from the ACCELERATE registry. Blood Adv. (2025) 9:1952–65. doi: 10.1182/bloodadvances.2024014391, PMID: 39951615 PMC12018988

[14] Pierson SK Bagg A Alapat D Lim MS Lechowicz MJ Srkalovic G . Characterization of castleman disease reveals patients with oligocentric adenopathy and clinicopathologic characteristics similar to unicentric castleman disease. Blood. (2021) 138:1622–2. doi: 10.1182/blood-2021-153840

[15] Beckham TH Yang JC Chau KW Noy A Yahalom J . Excellent outcomes with surgery or radiotherapy in the management of castleman disease including a case of oligocentric disease. Clin Lymphoma Myeloma Leukemia. (2020) 20:685–9. doi: 10.1016/j.clml.2020.05.002, PMID: 32522439 PMC7541423

[16] Van Rhee F Voorhees P Dispenzieri A Fossa A Srkalovic G Ide M . International, evidence-based consensus treatment guidelines for idiopathic multicentric Castleman disease. Blood. (2018) 132:2115–24. doi: 10.1182/blood-2018-07-862334, PMID: 30181172 PMC6238190

[17] Zhang L Liu QH Zhou H Zhang HL Dong YJ Wang XB . Asymptomatic multicentric Castleman disease: a potential early stage of idiopathic MCD. Blood Adv. (2024) 8:5598–602. doi: 10.1182/bloodadvances.2024013728, PMID: 39293084 PMC11541685

[18] Yu L Shi M Cai Q Strati P Hagemeister F Zhai Q . A novel predictive model for idiopathic multicentric castleman disease: the international castleman disease consortium study. Oncol. (2020) 25:963–73. doi: 10.1634/theoncologist.2019-0986, PMID: 32852137 PMC7648372

[19] Zhou X Wei J Lou Y Xu G Yang M Liu H . Salvage therapy with lenalidomide containing regimen for relapsed/refractory Castleman disease: a report of three cases. Front Med. (2017) 11:287–92. doi: 10.1007/s11684-017-0510-2, PMID: 28367597

[20] Lee FC Merchant SH . Alleviation of systemic manifestations of multicentric Castleman's disease by thalidomide. Am J Hematol. (2003) 73:48–53. doi: 10.1002/ajh.10310, PMID: 12701121

